# Arteriovenous malformations of the colon: A report of two cases and review of the literature

**Published:** 2017

**Authors:** Ghodratollah Maddah, Abbas Abdollahi, Omid Rouhbakhshfar, Shirin Taraz Jamshidi, Masoumeh Hassanpour

**Affiliations:** 1 Endoscopic and Minimally Invasive Surgery Research Center, Mashhad University of Medical Sciences, Mashhad, Iran.; 2 Surgical Oncology Research Center, Mashhad University of Medical Sciences, Mashhad, Iran.; 3 Student Research Committee, Faculty of Medicine, Mashhad University of Medical Sciences, Mashhad, Iran.; 4 Cancer Research Center, Mashhad University of Medical Sciences, Mashhad, Iran.

**Keywords:** Gastrointestinal Hemorrhage, Arteriovenous Malformations, Hemangioma, Cavernous.

## Abstract

**Background:**

Arteriovenous malformations are one of the most common vascular disorders of the colon. Vascular disorders present as painless, high-volume rectal bleeding.

**Case Presentation:**

This study elucidates two rare cases of vascular disorders that are diagnosed as angiodysplasia of the left colon and cavernous hemangioma of the colon and rectum. The chief complaint in two patients was rectorrhagia. The patients who were diagnosed of ulcerative colitis were treated with sulfadiazine and prednisone. Due to continuous bleeding, the patients were referred to the surgery department for operation. The patients underwent total proctocolectomy.

**Conclusion:**

We discuss the faults in the diagnosis and management of vascular disorders of the intestine.

**A**rteriovenous malformations are one of the most common vascular disorders of the large intestine (1). They are abnormal shunts between the arteries and veins without a capillary bed and are divided into two general categories of congenital and acquired. Acquired arteriovenous malformations often occur following trauma or surgical procedures (2, 3). Angiodysplasia and cavernous hemangioma are two types of arteriovenous malformations. Cavernous hemangioma is a rare congenital disorder which manifests early in life (1, 4). Angiodysplasia is a degenerative lesion which can be found at any age, and its incidence increases with age (1). Vascular disorders presents as painless high-volume rectal bleeding (1, 4). There are a few reports of vascular disorders of the intestine and rectum (5, 6).

## Case presentation

**Case 1**

A 23-year-old man was referred to surgical department with chief complaint of rectorrhagia. The patient had a history of presence of bright blood in the stool since 5 months old which occasionally occurred intermittently after excretion. In average, the patient received one blood transfusion every two months. For this reason, colonoscopy was performed for the patient at the age of 5 and 16, in which respectively a 1.5 and 1 cm rectal polyps were detected and resected. The first colonoscopy reported a juvenile polyp and the second one an inflammatory polyp. At the age of 23, the patient was hospitalized in the Internal Medicine ward complaining of severe lower gastrointestinal bleeding. Upper gastrointestinal endoscopy was normal.

A colonoscopy showed that the rectum and the left colon with the length of 50 cm had edematous fragile and hemorrhagic mucosa and was filled with blood vessels. Biopsy of the colon showed lymphocytic inflammation which confirmed ulcerative colitis. The patient was treated for 6 months with sulfasalazine and prednisolone. As bleeding continued the patient was referred to the surgery department for surgical operation. The patient had no history of admission or consumption of medicine. He was only anemic. Abdominal examination was normal. In the examination of blood, no coagulopathy was found. The liver tests were normal. Abdominal and pelvic ultrasonography was also normal. The patient underwent total proctocolectomy with mucosectomy and ileal pouch-anal anastomosis. In pathology, angiodysplasia of the left colon was reported. And now after 8 years, the patient has no symptoms. 

**Case 2**

A 24-year-old male patient with anemia and rectorrhagia was referred to surgical services. The patient had periodic rectal bright bleeding from the first days of his birth. Byage eight, he underwent hemorrhoidectomy surgery. When he was 15 years old, because of rectal bleeding, abdominal pain, weakness and lethargy, and consequently intensification of the bleeding and severe anemia he was hospitalized in the internal medicine ward. Ultrasound, scans and gastrointestinal endoscopy were performed on him.

**Figure 1:**
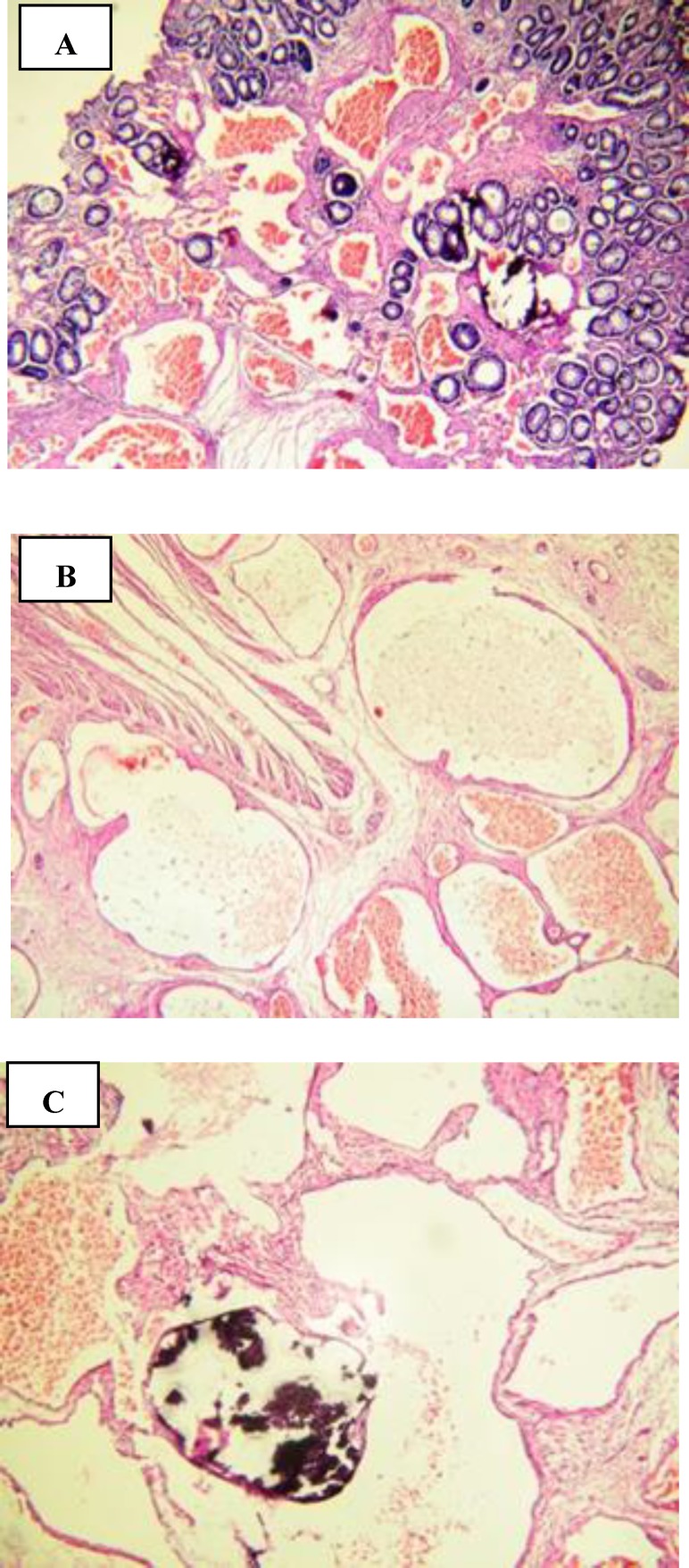
Cavernous hemangioma composed of large vessels with dilated lumen and thin walls with transmural involvement (A & B) and focal calcification (C

In ultrasonography, an enlarged spleen was reported. Aneurismal dilatation of the portal vein in the hepatic umbilicus as well as vascular torsion on the wall of the rectosigmoid was reported in color Doppler ultrasonography. Upper endoscopy demonstrated grade 2 esophageal varices. Biopsy of the liver (fine needle aspiration) revealed a non- cirrhotic liver. In colonoscopy, rectosigmoid mucosa showed edematous with petechial spots and vasodilatation. In the biopsy of rectal mucosa, lymphocytic inflammation was seen in submucosa and was suspicious to ulcerative colitis. The patient was treated with sulfadiazine and prednisone for 2 years since the diagnosis of ulcerative colitis and due to the continuous bleeding, he was referred to surgical services. In laboratory assays, microcytic anemia was the only abnormal finding. Upon the diagnosis of portal hypertension, the patient underwent splenectomy; four months later he underwent proximal splenorenal and mesocaval shunt procedure. Due to continuous bleeding, a laparotomy was performed in which hemangiomatous masses and vascular changes were identified in entire colon and terminal ileum. As a result, total proctocolectomy and anal mucosectomy were performed. During the operation, due to the resection of terminal ileum and shortage of the sigmoid, a sigmoid pouch was constructed for anastomosis. In pathology, cavernous hemangioma was reported (figure 1). And now after 4 years, the patient is asymptomatic.

## Discussion

The presented patients had a long history of rectal bleeding that was misdiagnosed with other causes of rectal bleeding. Moore et al. divided arteriovenous malformations of the gastrointestinal tract into three types: type 1 malformations include the arteriovenous malformations angiodysplasticdysplastic lesion) which occur in patients of 55 years or older. It is mainly seen in the right colon and lesions tend to be solitary so it was difficult to detect during operation. Type 2 lesions which occur in younger patients and with history of disease in the family are hereditary arterivenous malformations; the lesions are larger and mainly seen in the small intestine and can be diagnosed during surgery. Type 3 lesions, usually occur in patients with hereditary hemorrhagic telangiectasia (Osler–Weber–Rendu), and the lesions composed of multiple punctate angiomas (1, 7).

Options for evaluation of these patients include lower gastrointestinal (GI) endoscopy, abdominal x-ray imaging, barium enema and angiography (1, 4). Classic angiography is still the gold standard for the diagnosis of arteriovenous malformations (2, 3, 7). In other studies, computed tomography angiography and transrectal ultrasonography have been used to detect these lesions (2, 3). Other complementary diagnostic procedures such as upper GI endoscopy are better to rule out diffused gastrointestinal hemangiomatosis such as stomach and small intestine (9). Since non-intestinal involvements such as the pelvic cavity (8), spleen, liver, skin, and soft tissue may exist in these patients, the patients should be evaluated to rule out other syndromes such as Klippel Trenaunay (10, 11). 

One of the characteristic of wide lesions is its association with coagulation defects that can be presented with rectal bleeding or its exacerbation. Some patients have severe thrombocytopenia, hypofibrinogenemia and a decreased level of factor V and factor VIII (1). Differential diagnoses include malignant tumors, inflammatory bowel diseases and anorectal infection and diseases (12). Histologically, cavernous hemangioma includes numerous irregular blood-filled spaces which may spread to the mucosa, submucosa, muscle and serous membrane (12). The treatments of symptomatic intestinal vascular malformations are intravascular embolization (3) or surgical resection (13, 14). However, in cases of intravascular embolization, the recurrence rate is more common (3). In the case of rectal arteriovenous malformations, some authors suggest low anterior resection, total proctocolectomy with mucosectomy and ileal pouch (15, 16). It is better not to perform abdominoperineal resection in young patients (1). Another surgical method of vascular rectal lesions is coloanal sleeve anastomosis (17, 18).

Misdiagnosis is the major problem in these patients. Patients with chronic lower gastrointestinal bleeding should be evaluated for arteriovenous malformations. Treatment methods may vary from noninvasive procedures such as angiography and embolization to surgical resection of the lesion. We managed two cases with open surgery.

In conclusion, arterivenous malformations the rare cause of rectorragia and is usually presented with massive and chronic lower gastrointestinal bleeding which usually occur in the elderly, but may present in young adults.
